# 4th workshop purification therapies, from research to clinics “the end of the beginning”: executive summary

**DOI:** 10.3389/ti.2026.16711

**Published:** 2026-05-20

**Authors:** Mauro Atti, Giuseppe Remuzzi, Giuseppe Feltrin, Gabriel C. Oniscu, Robert J. Porte, Marco Ranieri, Mauro Rinaldi, Jean Louis Vincent

**Affiliations:** 1 Aferetica Srl, Bologna, Italy; 2 Istituto di Ricerche Farmacologiche Mario Negri IRCCS, Milan, Italy; 3 National Transplant Center, National Institute of Health (ISS), Rome, Italy; 4 Karolinska Institutet (KI), Huddinge, Sweden; 5 Department of Surgery, Erasmus MC Transplant Institute, Erasmus University Medical Center, Rotterdam, Netherlands; 6 University of Bari Aldo Moro, Bari, Italy; 7 Cardiac Surgery Division, Department of Surgical Sciences, Città della Salute e della Scienza University Hospital, University of Turin, Turin, Italy; 8 Hôpital Erasme-Cliniques universitaires de Bruxelles, Université libre de Bruxelles, Brussels, Belgium

**Keywords:** blood purification, cytokines adsorption, *ex-situ* perfusion, organ regeneration, perfusion machines

## Abstract

Over recent decades, advances in medicine have transformed the management of systemic diseases, severe inflammatory syndromes, and end-stage organ failure, expanding therapeutic possibilities in intensive care and transplantation. The fourth Workshop on Purification Therapies (WPT25), entitled “From Research to Clinic: The End of the Beginning”, marked an important moment in the maturation of extracorporeal blood purification therapies (EBPTs) and organ perfusion technologies. The first day focused on dysregulated inflammatory diseases and EBPTs, highlighting the role of inflammatory mediators as cytokines, pointed out the potentiality in their clinical applications in septic and cardiogenic shock. The discussion was focused on patient selection, timing, dosing, and drug–device interactions. The second day addressed organ preservation and regeneration, emphasizing *in situ* and *ex situ* perfusion strategies to expand donor eligibility—including DCD and extended criteria donors—while mitigating the iatrogenic effects as the ischemia–reperfusion injury. Discussions explored temperature management, inflammatory modulation during procurement and treatment, and future perspectives such as personalized perfusion protocols and xenotransplantation. With 550 participants, 26 oral presentations, practical workshops, and 161 scientific contributions published in one special issue of Transplant International, the meeting consolidated evidence and try to define priorities for integrating purification and perfusion therapies into clinical practice. Abstracts from the meeting are published in Transplant International: “Abstract Book of the 4th Workshop Purification Therapies From Research to Clinics “The End of the Beginning”, September 19th-20th, 2025.” at https://www.frontierspartnerships.org/research-topics/197/aferetica---wpt-2025-meeting-abstract.

Medicine has made extraordinary progress over the past decades, enabling the management and eradication of diseases that once represented a major burden for previous generations. These advances now allow us to treat systemic diseases and syndromes that until recently would have been considered terminal. This includes severe organ failure resulting from dysregulated inflammatory responses in critically ill patients, as well as organ failure that can only be resolved through transplantation.

Substantial progress has been achieved in both scientific knowledge and technological development in these fields, opening new clinical possibilities. Nevertheless, significant challenges remain in Transplant Medicine and Intensive Care, stimulating the development and progressive implementation of novel therapeutic strategies.

In this context, the fourth edition of the Workshop on Purification Therapies (WPT25) – *From Research to Clinic: “The End of the Beginning”* – aimed to contribute to scientific and clinical advancement in the development, validation, and implementation of new therapies in organ transplantation and dysregulated inflammatory diseases. The history of these meetings, initiated more than a decade ago, has progressively evolved from the discussion of exploratory concepts to structured clinical practice, supported by increasing mechanistic understanding and accumulated clinical experience.

The fourth edition marked not only the consolidation of previous achievements but also the transition toward a new phase of maturity, characterized by greater awareness, more in-depth clinical questions, and an increasingly international perspective.

Recognizing the significance of this moment, the workshop was subtitled *“Are We at the End of the Beginning?”* This question reflects the collective consideration of the scientific community: after years of pioneering work in organ perfusion technologies and hemoadsorption, the field has reached a stage where fundamental principles have been established, early clinical evidence has emerged, and initial uncertainties have been partially resolved. At the same time, this progress has revealed new challenges that require more rigorous investigation, higher-quality evidence, and careful integration into clinical pathways. The workshop was therefore conceived as a forum to assess the current state of the field and define priorities for its future development.

Among these, extracorporeal blood purification therapies (EBPTs) have gained increasing importance in intensive care practice. Despite their growing use, uncertainties persist regarding optimal indications and timing.

Concurrently, transplant medicine has expanded donor eligibility criteria to include marginal organs from donation after circulatory death (DCD) and extended criteria donors (ECD). Research has therefore focused on advanced strategies for organ preservation and treatment aimed at maintaining viability, enabling functional assessment, and allowing targeted therapeutic interventions prior to transplantation.

The first day of the workshop was dedicated to dysregulated inflammatory diseases and extracorporeal purification therapies. Particular attention was given to the complex and dynamic role of inflammatory mediators—both pro-inflammatory and anti-inflammatory cytokines—in the pathophysiology of dysregulated inflammatory syndromes.

Clinical experiences were presented demonstrating that hemoadsorption and other purification techniques, including when combined with standard treatments such as antimicrobial therapy, may contribute to the control of inflammatory mediators and provide advanced hemodynamic support. Special attention was given to the possible interactions that may occur when these therapies are used together, for example, between extracorporeal purification techniques and medications such as antibiotics. Understanding these interactions requires a thorough knowledge of the underlying mechanisms.

Discussions addressed the application of extracorporeal therapies not only in septic shock but also in cardiac surgery and cardiogenic shock, highlighting the versatility of these approaches while emphasizing the need, across all clinical scenarios, for careful patient selection as well as appropriate dosing and timing of interventions.

The second day focused on organ preservation and regeneration in transplantation, as well as future perspectives, including xenotransplantation.

Sessions explored advances in *ex situ* organ management, including *in situ* and *ex situ* perfusion, and their role in improving transplant quality, expanding the donor pool, and enabling therapeutic interventions on the organ prior to implantation.

These scientific sessions were complemented by practical workshops that allowed participants to interact directly with perfusion technologies, promoting operational understanding and facilitating knowledge transfer from experienced centres to those adopting these techniques.

Discussions also addressed the current state of solid organ transplantation. Experts examined when and how to implement organ perfusion, how to tailor approaches to different organs and donor types, and how emerging data may influence clinical guidelines. Particular attention was devoted to temperature management in order to optimally prevent ischemia–reperfusion injury and thermal stress to the organ.

A critical evaluation was also dedicated to the influence of inflammatory mechanisms during both organ procurement and organ treatment phases, highlighting how organs are retrieved, preserved, perfused, and treated in an environment burdened by inflammatory mediators, thereby providing a strong rationale for integrating purification strategies into existing clinical workflows.

The workshop concluded with a forward-looking perspective, including the potential for personalized perfusion protocols, organ-directed targeted therapies, and closer integration between extracorporeal technologies and immunomodulatory strategies, with the awareness that perfusion will likely be required even in areas that now appear increasingly within reach, such as xenotransplantation.

A key theme throughout the workshop was the importance of generating robust scientific evidence, recognizing that the clinical experience of thousands of centres worldwide can provide valuable practical insights.

The program included 26 oral presentations, three practical sessions involving more than 200 participants, and 161 scientific contributions in poster format—original works collected in this supplement that represent the true innovative output of the Workshop.

These contributions covered a broad spectrum of experimental, translational, and clinical experiences, highlighting ongoing challenges.

The 161 scientific contributions published in this special issue of *Transplant International Journal* were systematically organized and classified according to specific thematic areas to facilitate consultation and enable pragmatic use of the content by clinicians and researchers.

This special issue is intended not only as a report of the workshop but also as a resource reflecting the current state of the art in purification therapies applied to transplantation and dysregulated inflammatory diseases.

In summary, the fourth Workshop on Purification Therapies, with 550 participants from around the world, promoted interdisciplinary dialogue and, by emphasizing evidence-based practice, contributed to the advancement of perfusion and purification therapies in modern medicine.

